# Celiac Disease and Gluten-Free Diets: A Path or Barrier to Food (In)Security?

**DOI:** 10.3390/nu17121956

**Published:** 2025-06-08

**Authors:** Camila dos Santos Ribeiro, Claudia B. Pratesi, Renata Puppin Zandonadi

**Affiliations:** 1 Department of Nutrition, University of Brasília, Brasília 70910-900, Brazil; camilasribeiro15@gmail.com; 2 College of Population Health, University of New Mexico, Albuquerque, NM 87131, USA

**Keywords:** celiac disease, gluten-free diet, food security, food insecurity

## Abstract

**Background:** Celiac disease (CD) is an autoimmune condition triggered by gluten ingestion. The only effective treatment is adherence to a gluten-free diet (GFD), which is challenging due to the widespread presence of gluten in foods and the lack of physical and financial access to gluten-free options, among other factors that can lead to food nutrition insecurity (FNI). FNI, defined as the difficulty in accessing adequate food, is a factor that not only affects the need to adhere to a GFD but also compromises adherence itself. **Objective:** Review the scientific literature on the association between FNI, celiac disease, and adherence to a gluten-free diet. **Methodology:** This integrative review was conducted systematically using the PubMed, Scopus, and Web of Science databases, selecting studies that evaluated food security and insecurity among celiac patients. The keywords used with the Boolean operators were “celiac disease” AND/OR “gluten-free diet” AND “food insecurity” AND/OR “food security”. The search did not restrict language or geographic location, and studies were selected independently by two reviewers. **Results:** Ten publications met the inclusion criteria and were selected for the integrative review. FNI has been studied over the last five years in CD patients, but there is a lack of studies in different regions. FNI negatively affects the quality of life for those with CD and contributes to more severe symptoms and lower adherence to the GFD, especially in children and low-income families. Factors associated with higher risks of FNI in those with celiac disease include income, education, living in rural or non-central areas, and availability. These factors reinforce the negative impact of the association between FNI and adherence to the GFD in CD patients. **Conclusions:** The study of FNI in celiac individuals is a relatively recent development. The prevalence of FNI in this population is concerning and higher than in the general population, and it is associated with adherence to the GFD. Therefore, this topic demands extensive public policies to improve the health, quality of life, adherence, and treatment of CD patients.

## 1. Introduction

Celiac disease (CD) is a chronic autoimmune condition triggered after the ingestion of gluten (a protein complex that is naturally present in wheat, rye, and barley and their derivatives) by genetically predisposed individuals, which affects the small intestine and causes villous atrophy, manifesting in symptoms and clinical manifestations [1,2]. Studies estimate that CD affects between 0.7% and 1.4% of the world population. It is considered a public health problem, neglected and underdiagnosed due to a lack of knowledge about CD among the population and healthcare professionals, or insufficient diagnostic methods in some countries [3,4,5].

The CD treatment involves strict adherence to a gluten-free diet (GFD), which can reverse the damage caused to the intestinal mucosa and improve the quality of life for CD individuals [6,7,8]. Several factors are involved in adherence to the GFD, including the level of education, the patient’s perception and self-efficacy regarding the diet, knowledge about CD, duration of the GFD, instruction provided by qualified professionals, unconscious gluten consumption, the exact label for gluten-free products, difficulty in physical and economic access to gluten-free food [9,10]. Therefore, adhering to a GFD is particularly challenging due to insufficient guidance on healthy gluten-free meal preparation, the high cost of gluten-free products, the risk of gluten cross-contact, and social exclusion, among other factors. In this sense, a GFD can harm or be harmed by food insecurity resulting from socioeconomic conditions [11]. In this sense, there is a crucial role of sociodemographic factors in adherence to a GFD and GFDs impairing celiac individuals’ economic aspects [12,13].

Food nutrition insecurity (FNI) is related to the inability to access or significant risk in accessing adequate and sufficient food due to a lack of economic, social, or physical resources [14]. It can lead to a significant risk of decreased nutrient intake and adverse health consequences such as growth retardation, fatigue, and cognitive delays [15]. Thus, we can discuss the effect of FNI on adherence to CD treatment, since treatment also depends on acceptance and access to food, which is related to socioeconomic aspects.

Evaluating FNI involves several aspects, including diet quality, nutritional status, environment, climate, and income [16]. FNI assessment must be conducted at the population level to guide public policies precisely enough to support interventions targeted at households and individuals [17]. Several instruments have been developed to assess food nutrition security (FNS) [18]. Among them, the US Household Food Security Survey Module (USHFSSM) is notable, as it has been widely used since 1992, with cultural adaptations in various socioeconomic contexts [19,20,21]. Another notable instrument is the Food Insecurity Experience Scale Survey Module (FIES-SM), proposed in 2013 by the Food and Agriculture Organization (FAO) for application and validation in several countries [22]. Regardless of the type of instrument used, the FNI assessment stands out as important for identifying groups at risk and for supporting these groups in preventing negative health outcomes, thereby guaranteeing the human right to adequate food.

In this sense, CD individuals may be at an increased risk of food and nutrition insecurity due to the need for gluten-free foods in their diet, a lack of adequate access to these foods, and high prices despite access to quality food. Therefore, a GFD for CD individuals is considered a fundamental need to reduce symptoms and secondary health outcomes [23]. Given the complexity of CD treatment and the importance of accessing and consuming safe gluten-free food, it is essential to evaluate these issues to inform patients, researchers, food industries, and governments so that they can ensure the food security and health of individuals with CD. Therefore, this review aims to describe the association between FNI and GFD in CD.

## 2. Materials and Methods

This study is an integrative review using a systematic approach. The research was guided by the following question: What evidence is available about the association between food insecurity and a gluten-free diet in CD individuals?

The studies were selected from the following bibliographic databases: PubMed, Web of Science, Scopus, and Google Scholar (limited to the first 100 listed on this platform). The keywords used with the Boolean operators were “celiac disease” AND/OR “gluten-free diet” AND “food insecurity” AND/OR” food security.”

**Figure 1:**
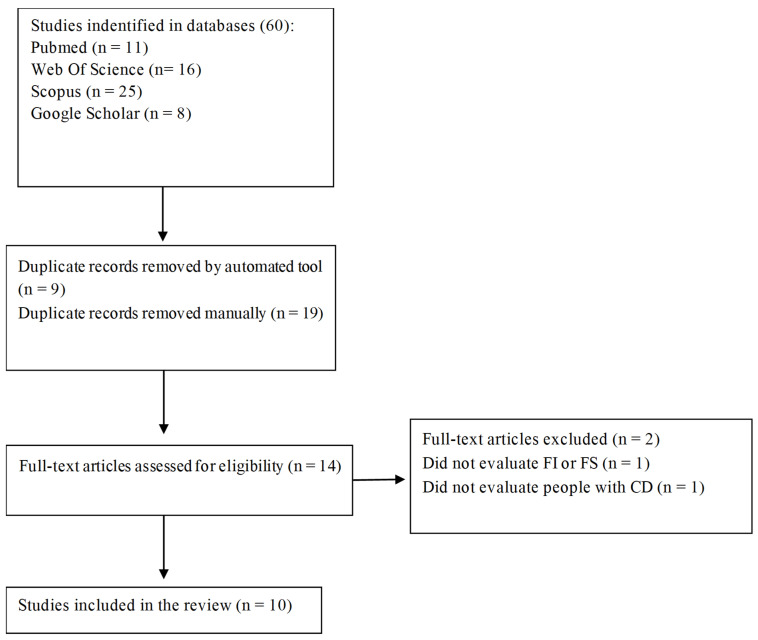
Flowchart of study selection process.

The inclusion criteria were studies involving individuals diagnosed with CD and studies on gluten-free diets that evaluated food insecurity or food security, from any geographic region, without restriction on language or year of publication. Web software was used to remove identical duplicates automatically, and duplicates not recognized by the software were manually removed by the reviewer. The selection process is described in Figure 1.

## 3. Results

### 3.1. Characterization of Studies

The database search yielded 60 studies. After removing duplicates and applying the inclusion criteria, ten publications were included [12,13,21,22,23,24,25,26,27,28] (Table 1). The two publications were treated as a single study, as they were based on the same study population [21,24]. It is evident that studies in this field are scarce, with no studies from countries from Latin America and Oceania, only three studies in Canada [13,21,24], two studies in the US [23,27], one study in Saudi Arabia [22], one in Jordan [12], one in Iran [26], one in the Republic of Moldova [28], one in the Netherlands [25]. Most studies were cross-sectional [12,13,21,24,25,26] (n = 8; 80%), and two were cohort studies [22,27].

Studies on FNI in CD are a recent and still underexplored subject, especially since all included studies were published within the last six years, with most published after the COVID-19 pandemic [12,13,21,22,23,24,27,28].

### 3.2. Instruments Used in the Studies

The studies used different instruments to evaluate the FNI: three of them used USDA 6-item short form of the Household Food Security Survey Module (HFSSM) [13,23,25], two used the USDA 18-item food security questionnaire [26,27], and two of them mentioned the use of the USDA food security questionnaire but did not mention the version used [21,24]; two used the Food Insecurity Experience Scale Survey Module (FIES-SM) [12,22]; two used the Hunger Vital Sign Screener [13,23]; and one used an algorithm to evaluate the countries on public policies related to FNS for people with CD [28]. Three studies evaluated FNI in children with celiac disease and five in adults with celiac disease (Table 1). Three studies adapted a tool that incorporated “gluten-free food” into each screening question of the Hunger Vital Sign Screener or the USDA 6-item short form of the HFSSM to assess GF food insecurity risk [13,23,25].

### 3.3. Food and Nutritional Security and Insecurity Among Celiac Patients

Considering all studies, the FNI frequency varied from 15% to 95% in the studied population (Table 2). In a study assessing FNI among 378 US CD patients using the Hunger Vital Sign Screener, 81 (21%) had FNI before the COVID-19 pandemic, and the FNI percentage increased to 27% during the pandemic [23]. In Canada, FNI affected 233 (47%) of the households surveyed using the same instrument, with over 30% reporting low to very low FNS [13].

The study conducted in the Netherlands with 548 celiac adults using the USDA 6-item HFSSM showed that 127 (23.2%) were facing FNI [25]. The Canadian study using the USDA 6-item HFSSM to evaluate FNS among 498 celiac children revealed that 50.7% of them experiencing high FNS (no reports of problems with access to food); 16.8%, moderate FNS (indicating anxiety about food sufficiency in the household); 17.9%, low FNS (indicating a reduction in the quality, variety, or desirability of the diet, with little or no indication of reduced food intake); and 14.6%, very low FNS (with reports of multiple indications of disrupted eating patterns and reduced food intake) [13]. A US study evaluating FNI among 378 CD patients reported using the Hunger Vital Sign Screener and the USDA 6-item HFSSM [23]. However, the authors did not present results from the USDA 6-item HFSSM application [23].

Using the USDA 18-item food security questionnaire, an Iranian study of 62 celiac children showed a mean food insecurity score of 3.4 ± 2.25, indicating insecurity without hunger (mild food insecurity) [26]. Of 62 Iranian celiac children, 30.6% were food-secure, 35.5% were food-insecure without hunger, 24.2% were food-insecure with mild hunger, and 9.7% were food-insecure with intense hunger (severe food insecurity) [26]. In the US, a study of 200 celiacs found that 15.9% (95% CI: 10.6%, 23.1%) lived in food-insecure households, 6.5% (95% CI: 3.2%, 12.9%) lived with marginal food security, 6.7% (95% CI: 4.1%, 10.9%) lived with low food security, and 2.7% (95% CI: 1.1%, 6.6%) lived with very low food security [27]. In a Canadian study evaluating 204 celiacs, FNI was identified in 17% of participants (5.5% were marginally FI; 8% were moderately FNI, and 3.5% were severely FNI) [24]. In this study, comparing celiac participants with FNS (n = 170), a greater proportion of celiacs facing FNI reported non-adherence to a GFD (76.5%) (26/34 vs. 70/170) [21,24].

In Saudi Arabia, a study using the FIES-SM questionnaire with 97 celiac patients showed that 38% (n = 37) of participants reported food security and 62% (n = 60) of reported food insecurity, with 29% reporting mild food insecurity, 22% medium, and 11% severe insecurity [22]. In Jordan, a study with 1162 celiac patients, using the same tool, showed 72.9% of participants were severely food-insecure, 12.2% were moderate, 3.7% were mild, and only 4.9% were food-secure [12]. A single study assessed the public policy situation for CD individuals and identified that the Republic of Moldova lacked adequate support and policies to ensure food security for CD individuals, as well as specific regulations for gluten-free foods [28].

Table 2 shows the frequency of FNS or FNI found in the studies, separated by the different tools used, and the frequency of gluten-free diet adherence among those facing FNS and FNI.

### 3.4. Factors Related to Food Insecurity in Celiac Disease

The studies assessed the socioeconomic levels of celiac patients and associated low income with a higher risk of FI [13,22,23,25,27]. Low income, poverty [adjusted odds ratio (aOR) = 0.30; 95% CI: 0.12, 0.75; *p* = 0.01], younger age [unadjusted OR = 1.26 per decade; 95% CI: 1.08, 1.47; *p* = 0.004), lower educational attainment (OR = 0.18; 95% CI: 0.06, 0.55; *p* = 0.004), and racial minority status were significantly associated with food insecurity among celiac patients [13,22,25,27]. Additionally, lack of homeownership and single parenthood were also factors [13].

Celiac patients predominantly live in rural areas [13,23], and small cities have higher rates of food insecurity [23]. In Saudi Arabia, individuals living in the central region were significantly more food-secure (n = 31, 84%) than patients residing in the non-central areas (n = 6, 16%) (*p* = 0.045) [22].

Cooking skills to prepare gluten-free foods (OR = 2.5; 95% CI = 1.5–4.3; *p* ≤ 0.001), social circumstances such as restaurants and friends (OR = 2.6; 95% CI = 1.1–6.4; *p* = 0.038), resources such as money and time to prepare gluten-free meals (OR = 2.5; 95% CI = 1.5–4.4; *p* = 0.001), and access to naturally gluten-free products (OR = 1.8; 95% CI = 1.0–3.1; *p* = 0.045) in diet were crucial factors associated with low food security and food insecurity, even when adjusted for age, income, and education [25].

Low availability and recent significant price increases in gluten-free products (*p* < 0.05) have affected FNI in Canadian celiacs [13]. A study reported the unavailability of gluten-free products in markets and difficulties in accessing gluten-free foods in restaurants and during travel, affecting FNS [17]. In addition, during the COVID-19 pandemic, intentional gluten intake increased due to the unavailability of gluten-free foods, and all households reported that availability had decreased (*p* < 0.001), impacting FNI [23].

**Figure 2:**
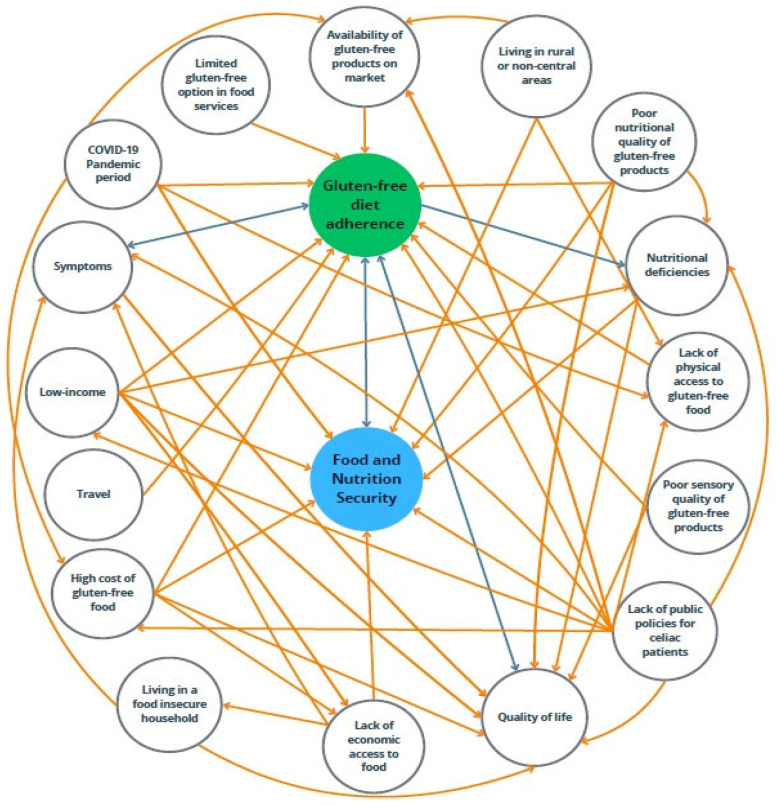
Factors influencing (and being influenced by) FNI and gluten-free diet adherence in celiac patients. Blue lines represent positive influences, and orange lines represent negative influences.

This review showed the association between FNI and GFD in CD. Figure 2 highlights the factors influencing (and being influenced by) FNI and gluten-free diet adherence in celiac patients and their complex relationship.

## 4. Discussion

This is the first review to address FNI and the gluten-free diet in CD patients. It is an important issue since, in CD, “food is the medicine” [29], and access to adequate, nutritious, and safe food is considered a human right that should be respected in all nations. Most studies included in this review were performed in Asia (n = 3; 30%) and North America (n = 3; 30%), followed by Europe (n = 2; 20%). No study was performed in South America, Africa, or Oceania, which is expected considering the estimates of the global CD prevalence in each region. A study showed a higher CD prevalence in Europe and Oceania (0.8%), followed by Asia (0.6%), Africa and North America (0.5%), and South America (0.4%) [3]. Therefore, we expected more studies in Europe and Oceania because of their higher prevalence of CD [3]. However, since Asia is one of the continents with the highest percentage of the population facing FNI (8.1%), it justifies the larger number of studies of FNI in the celiac population in this continent [30]. Despite no study evaluating FNI in the African CD population, this is the continent with the highest percentage of population facing FNI (20.4%), and it is probably the continent with the highest frequency of underdiagnosed CD in the population because of limited resources, lack of awareness among healthcare professionals and patients, and insufficient diagnostic facilities [30,31].

The prevalence of FNI among individuals with CD is consistently high, ranging from 23% to 72.9% across countries, with severe FNI averaging 25.8% [12,21,22,24,26]. In Iran, only 30% of children with CD were food-secure [26], while in Jordan, 72.9% experienced severe FNI—much higher than the 13.9% observed in the general population [32]. Similarly, in the Netherlands, 23.2% of adults faced FNI, and in Canada, 47% of celiac households were food-insecure, compared to 20.7% among the general adolescent population [21,24,25]. These findings highlight the heightened vulnerability of individuals with CD, underscoring the need for targeted public health interventions.

In Europe, there is a scarcity of studies on food insecurity, with only indirect data, and few studies in the countries of the continent, with FNI ranging from 5.5% to 43.3% in France and up to 9% to 100% in the United Kingdom in a study carried out with low-income families [33]. In Europe, FNI data are not routinely monitored as they are in Canada and the USA. This may be due to limited recognition of the concept, despite trends of increasing food insecurity after the 2008 crisis. In recent years, only a few studies have addressed this issue, as noted in a systematic review [33]. Although economically developed countries tend to have a lower FNI prevalence, economic growth alone does not ensure its elimination. In high-income nations, FNI can still be significant among vulnerable groups, especially where income inequality exists [17]. In the US, FNI is estimated to affect 11.7% of households (general population) [34], and between 15.9% and 27% of US CD people face FNI [23,27]. In Asia, data are even more limited, but Iran reported an FNI prevalence of 44.7% in children and 40.6% in adolescents [35], lower than that found in CD children (69.4%) [26]. In India, results vary greatly depending on the region of the population studied and the method used, with the FNI ranging from 8.9% to 99% in the general population [36].

The study of FNI in children becomes even more relevant, as ensuring safe and adequate access to CD treatment can prevent serious health consequences, such as short stature, muscle atrophy, anemia, and deficiencies in essential micronutrients that are crucial for growth [1,4,35]. FNI in CD children has been reported to be up to twice as prevalent as in the general population and is associated with lower quality of life and poorer adherence to a GFD [13]. Prevalence ranges from 27% to 69.4% among CD children, increasing the risk of intentional gluten consumption [23,26].

The results showed that FNI in CD is a recent subject and has been explored mainly during the COVID-19 pandemic period [12,13,21,22,23,24,25,26,27,28]. New studies from different countries may emerge, as these studies have highlighted the importance of evaluating CD from the perspective of food and nutrition security [12,13,21,22,23,24,25,26,27,28].

It is noteworthy that after the pandemic, there was an increase in studies on FNI, probably due to concerns about rising food prices, shortages, and loss of income, which may be particularly challenging for people accessing gluten-free foods, forcing people with CD to ingest gluten due to limited options [23,37]. Gluten ingestion with CD may cause several critical manifestations and adverse outcomes such as anemia, osteoporosis, cancer, infertility, liver disease, dysbiosis, and nutritional deficiencies [1,38,39]. Therefore, access to safe food for celiac patients is not just about overcoming hunger, but also about preventing severe clinical manifestations and reducing the risk of adverse health outcomes and mortality. These factors make celiac patients even more vulnerable to the effects of FNI, increasing morbidity [12]. Studies have shown that people with CD in an FNI situation have an increase in gastrointestinal symptoms and in the risk of comorbidities [21,24]. In this sense, evaluating the frequency of FNI in celiac patients and its outcomes may help the government outline public policies to face this serious public health problem.

The GFD includes eliminating gluten-containing and gluten-cross-contaminated foods and avoiding utensils and medications that may have been cross-contaminated. Although it may seem like a simple treatment, adopting and maintaining a GFD requires significant changes in eating habits and can be challenging due to practical and psychosocial barriers [40]. Barriers such as separating utensils between products that contain gluten and those that do not, maintaining a more rigorous cleaning routine for shared utensils to avoid gluten cross-contact, as well as using separate storage for ingredients and products represent an additional expense for people who need to adopt a gluten-free diet. This can impact the amount of food purchased, increasing the risk of FNI.

Unsurprisingly, all studies that assessed socioeconomic levels found that low income was associated with a higher risk of FNI [13,22,23,25,27]. Poverty, low educational levels, non-white and younger people, and those living in rural areas were associated with a higher frequency of FNI among people with CD [13,23,25,27]. Accessibility and availability of safe food are major concerns for families of children with CD, both those in food security and food insecurity [13]. A study showed that parents reduce other essential costs, such as their own food intake and leisure time, to cover the purchase of gluten-free foods (more expensive than their gluten-containing counterparts), regardless of their income [13]. Another study reports that CD people from lower socioeconomic groups cannot afford gluten-free foods, thus impacting their ability to adhere to the GFD and potentially leading to CD-related morbidity and additional healthcare costs, impairing the purchasing power for gluten-free products and entering a risk cycle [41].

FNI may pose a health risk by increasing the likelihood of intentional gluten ingestion due to difficulty in accessing gluten-free products, which are traditionally scarcer and more expensive. In a multicenter study in the United States, FNI was highly prevalent among CD patients and was associated with lower rates of GFD compliance and significant deficiencies in macronutrient and micronutrient intakes [27].

There is no specific validated instrument to assess FNI in CD people associated with the GFD. Three studies included in this review adapted their questions by including the term “gluten-free food” in each item to evaluate the burden of GFDs in FNI [13,23,25]. The USDA 18-item food security questionnaire is designed to assess food insecurity in the general population but was used without adaptation for celiacs [26,27] alongside the FIES-SM [12,22]. Despite the importance of using a general instrument that allows for the comparison of different populations, the use of a specific validated instrument to assess FNI directly related to the gluten-free diet would allow for a more accurate assessment and would favor the proposal of public policies to cope with FNI experienced by CD people. Furthermore, using a general instrument may underestimate the results, as CD people have specific dietary demands that compromise their household budget and nutritional status.

Health-related quality of life (HRQoL) is widely discussed and related to GFD adherence, but only these most recent studies have sought to relate this aspect to FNI [12,21,25]. In this sense, five studies observed a significant negative impact of GFD on quality of life and mental well-being, with greater depression and anxiety in those in FI [12,13,21,22,24,25]. Lack of food accessibility to meet safe, healthy, and nutritious needs leads to non-adherence to GFDs and is also a major barrier to HRQoL, particularly due to increased symptoms resulting from non-adherence to GFDs [12,21]. This is partly due to the challenges of finding safe gluten-free options and limited access to healthier substitutions [12,22]. Furthermore, studies were unanimous in pointing out that CD people in an FNI situation have worse adherence to GFD [12,13,21,24,27,41].

Although gluten-free foods have become more popular in recent years, they continue to be reported as more expensive and less readily available in high-cost markets than their gluten-containing counterparts. Hence, the financial burden of the diet remains one of the major causes of low adherence to GFDs. In the US, gluten-free foods can be 183% more expensive than wheat-based ones. Although these products have become popular recently, they are still not sufficiently accessible [29,31]. Lack of adequate access to GF foods and their high prices place CD patients at risk for FNI [26], and vulnerable families face a significant challenge in accessing healthy gluten-free food [11,30]. High dietary costs were among the factors mentioned in the studies, especially in low-income regions, placing individuals with this condition at higher risk of FNI [13,17]. Most households expressed concerns about the high cost of gluten-free foods [13].

Access to gluten-free foods is still limited in markets, mainly in developing countries. Despite the potential and greater availability of gluten-free foods online, they are still substantially more expensive than those in physical stores. Additionally, individuals with low socioeconomic status, those living in rural communities with limited internet access, and those with low digital literacy often face reduced access to gluten-free foods [13,26]. Gluten-free foods require advanced technology to maintain palatability, avoid sensory impairments, and remain nutritionally adequate, without altering perishability. These processes can increase the price of products, and product acceptance is still a challenge, generating rejection and increasing food waste [26,27,42]. Compared to their counterparts, industrialized gluten-free foods were classified as less healthy, as they have higher levels of fat, saturated fat, sugar, and salt and lower levels of protein and fiber [43], directly impacting the quality of food that people with CD consume and consequently their FNI.

Studies have shown that children with lower FNS were less likely to consume fruits and vegetables, while children facing FNI had worse dietary quality [13,25]. Furthermore, CD individuals facing FNI have more difficulty eating and cooking naturally gluten-free foods. Consumption of total calories, protein, and carbohydrates was significantly higher in CD patients with FNS than in those facing FNI [27], demonstrating the health hazards of CD people living in FNI.

One study assessed FNS through public policies implemented in the Republic of Moldavia and showed that the country lacks adequate political support to ensure food security for people with gluten-related disorders [28]. Appropriate social and economic policies for the adequate and safe production of gluten-free products, adequate prices, and greater access to them can help control CD patients’ FNI [26]. In addition, studies reinforce the importance of professionals considering socioeconomic and educational levels when evaluating and guiding people with CD, since the unavailability of food and the high diet cost are the most common reasons for non-adherence to a GFD.

## 5. Conclusions

Despite the limited number of studies exploring this theme, this review demonstrates the potential association between FNI and GFDs in CD. It highlights the importance and urgency of conducting further studies related to the impact of CD on FNI and the impact of FNI on the treatment of CD, since these data would encourage the formulation and implementation of public policies for people with CD to ensure their human right to a healthy and safe diet. FNI represents a significant risk and barrier to GFD, thus compromising the only effective treatment for CD. Studies show that social factors, such as income, education, low supply, and difficulty in physical and economic access to safe gluten-free foods, directly and negatively affect the quality of life of CD people and compromise their FNS. The lack of adequate access to GFDs is serious, as it impacts not only the intensification of symptoms, nutritional risk, and morbidities, but also highlights and perpetuates situations of exclusion and social vulnerability. Given this, public policies must ensure universal and equitable access to gluten-free foods, as well as constant multidisciplinary assistance for nutritional guidance and monitoring. Therefore, it is urgent to expand the debate and encourage scientific production on nutritional and food and nutrition (in)security, especially for low-income and vulnerable populations that depend exclusively on dietary treatment and safe and healthy foods to maintain their health.

## Figures and Tables

**Table 1:** Characterization of the studies.

Author	Country	Objective	Type of Study	Characterization and Number of Participants	Participants’ Age (Years)	Females (%)	FNI Assessment Tool	Main Results
Al- Sunaid et al. 2021 [22]	Saudi Arabia	To assess the association between adherence to a GFD, AI, and HRQoL in a cohort of individuals with CD living in Saudi Arabia.	Cohort	Adults (97)	34 ± 9	89%	Food Insecurity Experience Scale Survey Module (FIES-SM)	A total of 62% of participants faced FNI. Individuals living in non-central regions had higher levels of FNI. Gluten-free foods were not available at the supermarket for 53.55%. Additional challenges to accessing gluten-free foods included travel, lack of pita breads, and limited gluten-free options at restaurants. FNI significantly influences the adherence to a GFD and is associated with lower HRQOL in terms of both emotional well-being and mental health.
Ma et al., 2021 [27]	US	To systematically evaluate the impact of food security on patients with CD and its association with GFD adoption and nutritional intake in the United States.	Cohort	Adults (200)	42.1 (17.7 years)	60.4%	USDA 18-Item Standard Food Security Survey (SFSS)	A total of 15.9% of CD patients live in a food-insecure household. Patients who were food secure were significantly more likely to be on a GFD compared with patients who were food-insecure. FNI is associated with lower adherence to a GFD and significant deficiencies in macronutrient and micronutrient consumption.
Du et al., 2022 [23]	US	To better understand FNI in households with a child on a prescribed GFD and how food insecurity may affect GFD adherence during the pandemic.	Cross-sectional	378 households with CD children	7	61%	Hunger Vital Sign Screener and National Center for Health Statistics US Household 6-Item Short Form Food Security Survey Module (HFSSM)	In the pre-pandemic period, specifically about GF foods, 21% of the households were screened for FNI, and 27% were screened during the COVID-19 pandemic. All households reported that the availability of GF foods decreased during the pandemic (*p* < 0.001). Rural communities had the highest rates of food insecurity and GF FNI. GF food insecurity may pose a health risk by increasing the likelihood of intentional gluten ingestion.
Elsahoryi, N.A. et al., 2024 [12]	Jordan	To estimate the relationship between FNI and HRQoL in patients with CD and to assess whether this relationship is mediated or moderated by adherence to GFD.	Cross-sectional	1162 (adults)	28.52 ± 8.48	72.40%	Food Insecurity Experience Scale Survey Module (FIES-SM)	A total of 79.2% of CD patients in Jordan suffered from severe FNI and 4.9% presented FNS. There is a complicated interplay between FNI and HRQoL in patients with CD that is impacted by several factors, including gender, marital status, and income.
Khalifeh, et al., 2019 [26]	Iran	To evaluate the rate of food insecurity in children with CD and to assess its relationship with growth parameters in both genders.	Cross-sectional	62 (children)	11.04 (±3.8)	58.1%	USDA 18-item food security questionnaire	A total of 30.6% of patients were food-secure; 35.5% were insecure without hunger; 24.2%, insecure with mild hunger; and 9.7%, insecure with severe insecurity.
Leong, R. et al., 2023 [21] and Leong, R. et al., 2024 [24]	Canada	To evaluate the prevalence of FNI in patients with CD and to examine the relationship between FNI and GFD adherence, quality of life, and various gastrointestinal symptoms.	Cross-sectional	204 (adults)	-	-	Household Food Security Survey Module (HFSSM)	FI was identified in 17% of CD patients, and 3% were severely FI. FNI is a frequent problem in patients with CD, and it has a significant negative impact on adherence to treatment, symptom control, and quality of life.
Siminiuc, R., Turcanu, D., 2022 [28]	Republic of Moldova	To assess the level of care for people with celiac disease in the Republic of Moldova; in terms of public policies, to ensure a sustainable sector that effectively satisfies the food security of people with disorders associated with gluten consumption.	Cross-sectional	-	-	-	An algorithm was utilized, regarding global public policies in support of CD people	The Republic of Moldova does not have adequate policy support to ensure food security for people with gluten-related disorders, which poses major challenges and, as a result, may increase the complications of these problems.
Smeets, S. M. et al., 2024 [25]	Netherlands	To determine the prevalence of FNI among individuals with CD and non-celiac gluten sensitivity in the Netherlands and identify potential associations between food insecurity and diet quality.	Cross-sectional	548 (adults)	44.7 (±14.6)	90.30%	USDA 6-item Short Form of the Food Security Survey Module (HFSSM)	The overall prevalence of FNI was 23.2%. FNI participants were often younger, had a lower income and a lower educational level, and were associated with a significantly lower diet quality score compared with FNS participants. FNI participants were significantly more likely to experience difficulty with GF eating and cooking.
Wang, X. et al., 2024 [13]	Canada	To determine the prevalence and relevant household-level determinants of GF-FI in a multiethnic cohort of Canadian households with children with CD and the associations among child dietary adherence and changes in dietary quality on the GFD.	Cross-sectional	498 (children)			Hunger Vital Sign and USDA 6-item Short Form of the Household Food Security Survey Module (HFSSM)	A total of 47% of screened households were positive for GF-FI. The high prevalence of GF-FI in households with children with CD in this multiethnic cohort may negatively impact overall dietary quality and adherence to the GFD. Ongoing evaluation of the GF food environment and other factors influencing accessibility, affordability, and adequacy of the GFD is critical to forming effective policies to address these important issues.

CD, celiac disease; FIES-SM, Food Insecurity Experience Scale Survey Module; FNI, food and nutritional insecurity; HFSSM, Household Food Security Survey Module; HRQoL, Health-related Quality of Life; GFD, gluten-free diet; GF-FI, gluten-free food insecurity; NI, no information; USDA, United States Department of Agriculture.

**Table 2:** Frequency of food and nutrition security (FNS) or food and nutrition insecurity (FNI) in celiac patients and the frequency of gluten-free diet adherence among those experiencing FNS and FNI.

Country and Reference	Number of Participants	High FNS (%)	Moderate FNS (%)	Low FNS (%)	Very low FNS (%)	FNS (%)	FNI (%)	FNI Without Hunger (%)	FNI with Mild Hunger (%)	FNI with Intense Hunger (%)	Gluten-free Diet Adherence in FNS (n/Total; %)	Gluten-Free Diet Adherence Among FNI (n/Total; %)
Hunger Vital Sign Screener												
US [23]	378	N/A	N/A	N/A	N/A	76 *	24	N/A	N/A	N/A	N/A	N/A
Canada [13]	498	N/A	N/A	N/A	N/A	53	47	N/A	N/A	N/A	186/287; 91.6	101/234; 62.3%
Hunger Vital Sign Screener adapted to assess gluten-free food insecurity risk (incorporating “gluten-free food” in each screening question)												
US [23]	378	N/A	N/A	N/A	N/A	73 *	27	N/A	N/A	N/A	N/A	N/A
USDA 6-item HFSSM												
Netherlands [25]	548	N/A	N/A	12.8	10.4	76.8	23.2	N/A	N/A	N/A	N/A	N/A
Canada [13]	498	50.7	16.8	17.9	14.6	50.7 *	49.3 **	N/A	N/A	N/A	N/A	N/A
USDA 18-item food security questionnaire												
Iran [26]	62	N/A	N/A	N/A	N/A	30.6	69.4 **	35.5	24.2	9.7	N/A	N/A
US [27]	200		6.5	6.7	2.7	84.1 *	15.9	N/A	N/A	N/A	N/A	N/A
Canada [21] and [24]	204	N/A	N/A	N/A	N/A	83 *	17	5.5	8	3.5	100/170; 58.8%	8/34; 23.5%
FIES-SM												
Saudi Arabia [22]	97	N/A	N/A	N/A	N/A	38	62	29	22	11	32/37; 86.4%	39/60; 65%
Jordan [12]	1162	N/A	N/A	N/A	N/A	4.9	95.1	3.7	12.2	79.2	4/57; 0.34%	89/1105; 7.6%

* Data calculated from the sum of results that represents FNS, or by the difference of 100% and % of FNI; ** data calculated from the sum of results that represents FNI; the values were estimated based on the observed prevalence and the Pearson correlation between the variables (r = 0.489), assuming proportionality between the groups.

## Data Availability

Not applicable.

## Abbreviations

The following abbreviations are used in this manuscript:

CD	Celiac disease
FNI	Food and nutritional insecurity
FNS	Food and nutritional security
GFD	Gluten-free diet

## References

[1] Al-Toma A., Volta U., Auricchio R., Castillejo G., Sanders D.S., Cellier C., Mulder C.J., Lundin K.E.A. (2019). European Society for the Study of Coeliac Disease (ESsCD) guideline for Coeliac Disease and Other Gluten-Related Disorders. United Eur. Gastroenterol. J..

[2] Fasano A., Catassi C. (2012). Clinical Practice Celiac Disease. N. Engl. J. Med..

[3] Singh S., Singh P., Arora A., Strand T.A., Leffler D.A., Catassi C., Green P.H., Kelly C.P., Ahuja V., Makharia G.K. (2018). Global Prevalence of Celiac Disease: Systematic Review and Meta-Analysis. Clin. Gastroenterol. Hepatol..

[4] Sahin Y. (2021). Celiac Disease in Children: A Review of the Literature. World J. Clin. Pediatr..

[5] Taraghikhah N., Ashtari S., Asri N., Shahbazkhani B., Al-Dulaimi D., Rostami-Nejad M., Rezaei-Tavirani M., Razzaghi M.R., Zali M.R. (2020). An Updated Overview of Spectrum of Gluten-Related Disorders: Clinical and Diagnostic Aspects. BMC Gastroenterol..

[6] Bernardo D., Peña A.S. (2012). Developing Strategies to Improve the Quality of Life of Patients with Gluten Intolerance in Patients with and without Coeliac Disease. Eur. J. Intern. Med..

[7] Galli G., Esposito G., Lahner E., Pilozzi E., Corleto V.D., Di Giulio E., Aloe Spiriti M.A., Annibale B. (2014). Histological Recovery and Gluten-Free Diet Adherence: A Prospective 1-Year Follow-up Study of Adult Patients with Coeliac Disease. Aliment. Pharmacol. Ther..

[8] Wieser H., Ruiz-Carnicer Á., Segura V., Comino I., Sousa C. (2021). Challenges of Monitoring the Gluten-Free Diet Adherence in the Management and Follow-Up of Patients with Celiac Disease. Nutrients.

[9] Fernández Miaja M., José J., Martín D., Treviño S.J., Suárez González M., Bousoño García C. (2021). Study of Adherence to the Gluten-Free Diet in Coeliac Patients. An. Pediatr. (Engl. Ed.).

[10] Villafuerte-Galvez J., Vanga R.R., Dennis M., Hansen J., Leffler D.A., Kelly C.P., Mukherjee R. (2015). Factors Governing Long-Term Adherence to a Gluten-Free Diet in Adult Patients with Coeliac Disease. Aliment. Pharmacol. Ther..

[11] Lambert K., Ficken C. (2016). Cost and Affordability of a Nutritionally Balanced Gluten-Free Diet: Is Following a Gluten-Free Diet Affordable?. Nutr. Diet..

[12] Elsahoryi N.A., Ibrahim M.O., Alhaj O.A. (2024). Adherence to the Gluten-Free Diet Role as a Mediating and Moderating of the Relationship between Food Insecurity and Health-Related Quality of Life in Adults with Celiac Disease: Cross-Sectional Study. Nutrients.

[13] Wang X., Anders S., Jiang Z., Bruce M., Gidrewicz D., Marcon M., Turner J.M., Mager D.R. (2024). Food Insecurity Impacts Diet Quality and Adherence to the Gluten-Free Diet in Youth with Celiac Disease. J. Pediatr. Gastroenterol. Nutr..

[14] Peng W., Berry E.M. (2018). The Concept of Food Security.

[15] Gallegos D., Eivers A., Sondergeld P., Pattinson C. (2021). Food Insecurity and Child Development: A State-of-the-Art Review. Int. J. Env. Res. Public. Health.

[16] Fanzo J., Haddad L., Schneider K.R., Béné C., Covic N.M., Guarin A., Herforth A.W., Herrero M., Sumaila U.R., Aburto N.J. (2021). Viewpoint: Rigorous Monitoring Is Necessary to Guide Food System Transformation in the Countdown to the 2030 Global Goals. Food Policy.

[17] Gallegos D. (2025). Effects of Food and Nutrition Insecurity on Global Health. N. Engl. J. Med. Rev..

[18] FAO, IFAD, WHO, UNICEF, WFP (2024). The State of Food Security and Nutrition in the World 2024—Financing to End Hunger, Food Insecurity and Malnutrition in All Its Forms.

[19] Wehler C.A., Scott R.I., Anderson J.J. (1992). The Community Childhood Hunger Identification Project: A Model of Domestic Hunger—Demonstration Project in Seattle, Washington. J. Nutr. Educ..

[20] Segall-Corrêa A.M., Marin-León L., Melgar-Quiñonez H., Pérez-Escamilla R. (2014). Refinement of the Brazilian Household Food Insecurity Measurement Scale: Recommendation for a 14-Item EBIA. Rev. De Nutr..

[21] Leong R., Tandon S., Khaouli M., Blom J., Daca R., Verdu E., Armstrong D., Pinto-Sanchez M. (2023). The impact of food insecurity on adherence to a gluten-free diet in the adult celiac disease population attending a dedicated celiac clinic. Proc. Int. J. Psychol..

[22] Al-sunaid F.F., Al-homidi M.M., Al-qahtani R.M., Al-ashwal R.A., Mudhish G.A., Hanbazaza M.A., Al-zaben A.S. (2021). The Influence of a Gluten-Free Diet on Health-Related Quality of Life in Individuals with Celiac Disease. BMC Gastroenterol..

[23] Du N., Mehrotra I., Weisbrod V., Regis S., Silvester J.A. (2022). Brief Communication: Survey Based Study on Food Insecurity during COVID-19 for Households with Children on a Prescribed Gluten-Free Diet. Am. J. Gastroenterol..

[24] Leong R., Tandon S., Khaouli M., Blom J.-J., Daca R., Verdu E.F., Armstrong D., Sanchez M.I.P. (2024). Su1338 food insecurity negatively impacts gluten-free diet adherence and is associated with persistent symptoms in adult patients with celiac disease. Proc. Gastroenterol..

[25] Smeets S.M., Jong J.C.K., van der Velde L.A. (2024). Food Insecurity and Other Barriers to Adherence to a Gluten-Free Diet in Individuals with Celiac Disease and Non-Celiac Gluten Sensitivity in the Netherlands: A Mixed-Methods Study. BMJ Open.

[26] Khalifeh F., Riasatian M.S., Ekramzadeh M., Honar N., Jalali M. (2019). Assessing the Prevalence of Food Insecurity among Children with Celiac Disease: A Cross-Sectional Study. J. Food Secur..

[27] Ma C., Singh S., Jairath V., Radulescu G., Ho S.K.M., Choi M.Y. (2022). Food Insecurity Negatively Impacts Gluten Avoidance and Nutritional Intake in Patients With Celiac Disease. J. Clin. Gastroenterol..

[28] Siminiuc R., Ṭurcanu D. (2022). Food Security of People with Celiac Disease in the Republic of Moldova through Prism of Public Policies. Front. Public. Health.

[29] Catassi C., Chirdo F.G. (2025). The Gluten-Free Diet: The Road Ahead. Nutrients.

[30] Palmquist A., WHO, FAO, IFAD, UNICEF, WFP Hunger Numbers Stubbornly High for Three Consecutive Years as Global Crises Deepen: UN Report. https://www.who.int/news/item/24-07-2024-hunger-numbers-stubbornly-high-for-three-consecutive-years-as-global-crises-deepen--un-report.

[31] Gatti S., Rubio-Tapia A., Makharia G., Catassi C. (2024). Patient and Community Health Global Burden in a World with More Celiac Disease. Gastroenterology.

[32] Olaimat A.N., Alshami I.K., Al Hourani H., Sarhan W., Al-holy M., Abughoush M., Al-awwad N.J., Hoteit M., Al-jawaldeh A. (2022). Food Insecurity, Dietary Diversity, and Coping Strategies A Cross-Sectional Study. Nutrients.

[33] Zaçe D., Di Pietro M.L., Caprini F., de Waure C., Ricciardi W. (2020). Prevalence and Correlates of Food Insecurity among Children in High-Income European Countries. A Systematic Review. Ann. Ist. Super. Sanità.

[34] Nikolaus C.J., An R., Ellison B., Nickols-Richardson S.M. (2020). Food Insecurity among College Students in the United States: A Scoping Review. Adv. Nutr..

[35] Arzhang P., Abbasi S.H., Sarsangi P., Malekahmadi M., Nikbaf-Shandiz M., Bellissimo N., Azadbakht L. (2022). Prevalence of Household Food Insecurity among a Healthy Iranian Population: A Systematic Review and Meta-Analysis. Front. Nutr..

[36] McKay F.H., Sims A., van der Pligt P. (2023). Measuring Food Insecurity in India: A Systematic Review of the Current Evidence. Curr. Nutr. Rep..

[37] Amirian P., Zarpoosh M., Moradi S., Jalili C. (2023). Celiac Disease and COVID-19 in Adults: A Systematic Review. PLoS ONE.

[38] Itzlinger A., Branchi F., Elli L., Schumann M. (2018). Gluten-Free Diet in Celiac Disease—Forever and for All?. Nutrients.

[39] Ludvigsson J.F., Leffler D.A., Bai J.C., Biagi F., Fasano A., Green P.H.R., Hadjivassiliou M., Kaukinen K., Kelly C.P., Leonard J.N. (2013). The Oslo Definitions for Coeliac Disease and Related Terms. Gut.

[40] Möller S.P., Hayes B., Wilding H., Apputhurai P., Tye-Din J.A., Knowles S.R. (2021). Systematic Review: Exploration of the Impact of Psychosocial Factors on Quality of Life in Adults Living with Coeliac Disease. J. Psychosom. Res..

[41] Hanci O., Jeanes Y.M. (2019). Are Gluten-Free Food Staples Accessible to All Patients with Coeliac Disease?. Frontline Gastroenterol..

[42] Lee A.R., Wolf R.L., Lebwohl B., Ciaccio E.J., Green P.H.R. (2019). Persistent Economic Burden of the Gluten Free Diet. Nutrients.

[43] Fry L., Madden A.M., Fallaize R. (2018). An Investigation into the Nutritional Composition and Cost of Gluten-Free versus Regular Food Products in the UK. J. Hum. Nutr. Diet..

